# Supplementary material for: The tangled ways to classify games: A systematic review of how games are classified in psychological research
Source: PLoS One. 2024 Jun 24;19(6):e0299819. doi: 10.1371/journal.pone.0299819 (PMC11195997; doi:10.1371/journal.pone.0299819)
Supplement: S1 Table — (DOCX) [file pone.0299819.s001.docx]

Table S1. A table presenting in detail the genres used in individual papers

| Authors | SPORT | RPG | STRATEGY | SIMULATION | PUZZLE | DRIVING/  RACING | MMORPG | FPS | FIGHTING | ACTION-ADVENTURE | ACTION | PLATFORM | SHOOTER | ADVENTURE | RTS | RHYTHM/  MUSIC | MOBA | BOARD AND CARDS | SOCIAL/CASUAL | ARCADE | TBS | EDUCATIONAL | BROWSER | PARTY | MANAGEMENT | ACTION RPG | SURVIVAL/HORROR | SANDBOX | BEAT ‘EM ; UP | OTHER |
| --- | --- | --- | --- | --- | --- | --- | --- | --- | --- | --- | --- | --- | --- | --- | --- | --- | --- | --- | --- | --- | --- | --- | --- | --- | --- | --- | --- | --- | --- | --- |
| Abdullah et al. (2015) | X | X | X | X | X |  |  |  |  | X |  |  |  | X |  |  |  |  |  |  |  |  |  |  |  |  |  |  |  | Beat |
| Alonso-Diaz, Yuste-Tosina, Mendo-Lázaro (2019) | X | X | X |  | X | X | X |  |  | X | X | X | X |  | X |  |  |  |  |  |  |  |  |  |  |  |  |  |  | GRAPHIC ADVENTURE GAMES,  MAZES, EDUCATIONAL BOARD GAMES, BAT AND BALL GAME |
| Amiriparian er al. (2019) |  |  |  | X |  | X |  |  | X |  | X | X | X |  |  |  |  |  |  | X |  |  |  |  |  |  |  |  |  | WORLD BUILDING |
| Appel (2012) | X | X | X | X |  | X |  | X |  | X |  |  |  |  |  |  |  |  | X |  |  |  |  |  |  |  |  |  |  | PERLOR GAMEACTIVITY GAME |
| Azzizi et al. (2018) | X | X |  |  | X |  |  | X |  | X | X |  |  |  | X |  | X |  |  |  | X |  |  |  |  | X |  |  |  | NON-ACTION RPG, TB RPG, FANTASY GAMES |
| Balakrishnan and Griffiths (2019) | X | X | X |  | X | X |  |  |  |  | X |  |  | X |  |  |  | X | X | X |  |  |  |  |  |  |  |  |  | CASINO, TRIVIA, WORD |
| Bilginer et al.. (2021) | X | X | X | X |  | X |  |  | X | X |  |  | X |  |  |  |  | X |  | X |  |  |  |  |  |  |  |  |  | QUIZ/TRIVIA |
| Boric and Strauss (2021) | X | X | X | X | X | X |  |  | X |  | X | X | X | X |  | X |  |  |  | X |  |  |  | X |  |  |  |  |  | HEATLH & FITNESS |
| Bowman and Chang (2023) | X | X |  | X | X | X | X | X | X | X |  |  |  |  |  | X | X | X |  |  |  |  |  |  |  |  |  |  |  | CASINO/GAMBLING, VR, AR, OPEN WORLD |
| Braun et al. (2016) |  | X | X | X |  |  |  | X |  |  | X |  |  |  | X |  |  |  |  |  |  |  |  |  |  |  |  |  |  | RBS, life simulation |
| Casale et al. (2022) | X |  |  |  |  |  | X | X |  |  |  |  |  |  | X |  | X |  |  |  |  |  |  |  |  |  |  |  |  | X |
| Cho et al. (2017) | X |  |  |  |  | X | X | X |  |  | X |  |  |  | X |  |  |  |  |  |  |  |  |  |  |  |  |  |  | SMARTPHONE GAME |
| Choi et al. (2019) | X | X | X | X | X |  |  |  |  | X | X |  |  | X |  | X |  | X |  |  |  |  |  |  |  |  |  |  |  |  |
| Collins and Cox (2014) | X | X | X | X |  |  | X | X | X |  | X |  |  | X |  |  |  |  | X |  |  |  |  | X |  |  |  |  |  |  |
| Deleuze et al. (2017) |  |  |  |  |  |  | X | X |  |  |  |  |  |  |  |  | X |  |  |  |  |  |  |  |  |  |  |  |  |  |
| Demisova et al. (2019) | X | X | X |  | X |  | X |  | X | X |  | X | X | X |  |  | X |  |  |  | X |  |  |  | X | X | X |  | X | EROGE  ROGUELIKE  STEALTH  TACTICAL RPG  VEHICLE SIMULATION |
| Dickmeis and Roe (2019) | X | X | X | X | X | X | X |  | X |  |  | X | X | X |  |  |  |  |  |  |  |  |  |  |  |  |  |  |  |  |
| Dieris-Hirche et al. (2020) | X |  | X | X |  |  | X | X |  |  |  |  |  | X |  |  |  |  |  |  |  |  |  |  |  |  |  |  | X | X  JUMP ‘N’ RUN, |
| Dindar (2018) | X | X | X |  |  |  |  |  |  | X |  |  | X |  |  |  |  |  |  |  |  |  |  |  |  |  |  |  |  | BRAIN AND SKILL GAMES, |
| Dobrowolski et al. (2015) |  | X |  |  | X | X |  | X | X |  |  | X |  | X |  |  | X |  |  |  | X |  |  |  |  |  |  |  |  |  |
| Donati et al. (2015) | X | X |  |  |  |  | X |  | X |  | X | X |  |  | X |  |  |  | X | X |  |  | X |  | X |  |  |  |  | RETRO INDIE |
| Elliott et al. (2012a) | X | X | X |  | X | X | X | X |  | X |  | X | X |  | X | X |  | X |  |  |  |  |  |  |  |  |  |  |  | X  GAMBLING |
| Elliott et al. (2012b) | X | X | X |  | X | X | X | X |  | X |  | X | X |  | X | X |  | X |  |  |  |  |  |  |  |  |  |  |  | X  GAMBLING |
| Entwistle et al. (2020) | X | X | X | X | X | X |  |  | X | X | X |  | X |  |  |  |  |  |  |  |  |  |  |  |  |  |  |  |  | GLOBAL POSITIONING SYSTEM (Pokemon Go) |
| Floros and Siomos (2012) | X | X | X | X |  | X | X |  | X |  |  | X |  |  |  | X |  |  |  |  |  |  | X |  | X |  |  |  |  | FLIGHT SIM MOBILE/HANDFELDS THOUGHT |
| Fuster et al. (2016) | X | X | X |  | X |  | X |  |  |  | X |  |  |  |  |  | X |  |  |  |  |  |  |  |  |  |  |  |  |  |
| Gabbiadini and Greitemeyer (2017) |  |  | X |  | X | X |  |  |  |  |  | X |  |  |  |  |  |  |  |  |  |  |  |  |  |  |  |  |  | LOGIC, VIOLENT |
| Gaxkenbach et al. (2016) | X | X | X | X | X | X | X | X | X |  |  |  |  | X | X | X |  | X | X |  |  |  |  |  |  |  |  |  |  |  |
| Gilbert et al. (2018) | X |  |  |  |  |  |  |  |  |  | X |  |  |  |  |  |  |  |  |  |  |  |  |  |  |  |  |  |  | ONLINE GAMES |
| Green et al. (2017) | X |  |  | X | X | X |  |  | X | X |  |  |  |  | X | X | X |  | X |  | X |  | X |  |  | X |  |  |  | X  ACTION FPS, ACTION TPS, NON-ACTION, TURN-BASED, RPG, FANTASY |
| Guzsvinecz and Szűcs (2023) | X | X | X | X | X | X |  |  |  |  | X |  |  | X |  |  |  | X | X |  |  |  |  |  |  |  |  |  |  | EXPERIMENTAL |
| Han et al. (2020) | X | X |  | X |  |  |  |  |  |  | X |  | X |  |  |  | X |  |  | X |  |  |  |  |  |  |  |  |  |  |
| Hazel et al. (2022) |  | X | X | X | X | X | X | X | X |  |  |  |  |  |  | X | X |  | X |  |  |  |  |  |  |  | X |  |  | HIDDEN OBJECT, POINT AND CLICK, PUZZLE-ACTION TEXT, ADVENTURE TPS |
| Homer et al. (2012) | X |  |  | X | X |  | X | X | X |  |  |  |  |  | X |  |  |  |  |  | X |  | X | X |  |  |  |  |  | VIRTUAL LIFE WORLD 2D/SIMPLE |
| Howe and Cionea (2021) | X | X | X |  |  |  | X | X |  |  |  |  |  |  |  |  |  |  | X |  |  |  |  |  |  |  |  |  |  | RETRO |
| Jang and Byon (2020) | X |  |  |  |  |  |  | X |  |  |  |  |  |  | X |  | X |  |  |  |  |  |  |  |  |  |  |  |  | IMAGINATION, PHYSICAL, ENACTMENT – CCS, Hero-Shooting, BATTLE ROYALE, SPORT SIMULATION |
| Jiang and Zheng (2023) | X | X | X | X | X | X |  |  | X |  |  | X | X | X |  | X |  |  |  | X |  |  |  |  |  |  |  |  |  | IDNIE, PINBALL, QUIZ/TRIVIA |
| Jiwal et al. (2022) |  | X |  |  | X |  |  |  |  |  |  |  |  | X | X |  | X |  |  |  |  |  |  |  |  |  |  |  |  | X  ERG, MTG |
| Johnson et al (2012) | X | X | X |  |  | X | X | X | X | X |  |  | X |  | X |  |  |  |  |  | X |  |  |  |  | X |  |  |  |  |
| Johnson et al. (2016) | X | X |  | X | X | X | X |  | X | X |  | X | X |  | X |  | X |  |  |  | X |  |  |  |  |  |  |  |  |  |
| Kim et al. (2022) | X | X |  |  |  | X |  | X |  |  |  |  |  |  | X |  |  |  |  | X |  |  |  |  |  |  |  |  |  |  |
| Kiraly et al. (2022) | X | X | X | X | X |  | X | X |  | X |  | X | X |  | X |  | X | X |  |  | X |  | X |  |  |  |  |  |  | X  BATTLE ROYALE, TPS, AUTO, CHESS, BATTLE ARENA |
| Kühn and Gallinat (2013) |  | X |  | X | X | X | X |  |  | X |  | X |  |  |  |  |  |  |  | X |  |  |  |  |  | X |  |  |  | BUILDING  BALL  CLICK AND POINT  EGO SHOOTER  TPS |
| Laffan et al. (2016) | X | X |  |  | X |  | X |  | X | X |  |  |  |  |  |  | X |  |  |  |  | X |  |  |  |  |  | X |  | SHOOT ‘EM’ UP |
| Lange, Wühr and Schwartz (2021) | X | X | X | X | X |  |  | X |  | X | X | X |  | X |  | X |  |  | X |  |  | X |  |  |  |  |  |  | X | EROTIC, TPS, OPEN-WORLD, QUIZ, SHOOT ‘EM’ UP, WESTERN |
| Lee et al. (2014) | X | X | X | X | X | X |  |  | X | X | X |  | X |  |  |  |  |  |  |  |  |  |  |  |  |  |  |  |  |  |
| Lemmens and Hendriks (2016) | X | X | X | X | X | X |  |  | X | X |  |  | X |  |  |  |  |  |  |  |  |  |  |  |  |  |  |  |  |  |
| Li, Mills, Nower (2019) | X | X | X | X |  |  | X |  |  |  | X |  | X |  |  |  | X | X |  |  |  |  |  |  |  |  |  |  |  | BATTLE ROYALE |
| Li (2020) | X | X | X | X |  | X | X |  | X |  |  | X | X |  | X | X |  | X |  |  | X | X |  |  | X | X | X |  |  | ARCADE SHOOTER, ROGUE CHARACTER, ACTION CLASSIC, STRATEGY, RPG, TOP-DOWN SHOOTER, INTERACTIVE FICTION, TOWER DEFENCE, PARKOUR, EXPLORATION, |
| Li and Zhang (2020) |  | X | X | X | X |  |  |  |  |  |  |  | X |  |  |  |  |  |  | X |  |  |  |  |  |  |  |  |  |  |
| Lloyd, Frost, Jones (2019) | X | X | X |  |  |  |  | X | X |  |  |  |  | X |  | X |  |  | X |  |  |  |  |  |  |  |  |  |  | QUIZ |
| Lonergan and Weber (2019) | X | X | X | X | X | X |  |  | X | X |  |  | X |  |  |  | X | X |  | X |  |  |  |  |  |  |  |  |  | X  QUIZ/TRIVIA |
| López-Fernández et al. (2021) | X | X | X | X | X |  |  |  | X |  |  |  |  | X |  |  |  |  |  |  |  |  |  |  | X |  |  |  |  | ACTION, SHOOTER |
| Mandryk and Birk (2017) | X | X | X | X | X | X | X | X |  |  | X | X |  | X |  | X | X |  | X |  |  |  |  |  |  |  |  |  | X |  |
| Manero et al. (2016) | X |  |  | X |  | X | X | X | X |  |  |  |  |  |  | X |  |  | X |  |  |  | X |  |  |  |  |  |  | ADVENTURE & THRILLERS INVENTION/COGNITIVE |
| Männiko et al. (2017) | X |  | X |  |  | X | X | X |  |  |  |  | X |  |  |  | X |  | X |  |  |  |  |  | X |  |  |  |  | SOLO |
| Mathews et a. (2018) | X | X |  |  | X | X | X | X | X | X | X | X |  |  | X |  |  | X |  |  | X |  |  |  |  |  | X |  |  | GAMBLING |
| Mazurek and Engelhardt (2013) | X | X | X | X | X | X |  | X | X | X | X | X |  | X |  | X |  |  |  |  |  | X |  |  |  |  |  |  |  | MINI GAME |
| Mazurek et al. (2015) | X | X | X | X | X | X |  | X | X | X |  | X |  |  |  | X |  |  |  |  |  |  |  | X |  |  |  | X |  |  |
| McMahon, Wyeth and Johnson (2013) | X | X | X | X | X |  |  |  |  |  | X |  |  | X |  |  |  |  |  |  |  |  |  |  |  |  |  |  |  | BUILDING AND RESEOURCE GAMES |
| Mitchell et al. (2015) |  |  |  | X |  |  |  |  |  |  | X |  |  | X |  |  |  |  |  |  |  | X |  |  |  |  |  |  |  |  |
| Moffat et al. (2017) |  |  |  |  | X |  |  | X |  |  |  |  |  |  |  |  |  |  |  |  |  |  |  |  |  |  |  | X |  |  |
| Musetti et al. (2019) |  |  |  | X |  |  | X | X |  |  |  |  |  |  | X |  | X |  |  |  |  |  | X |  |  |  |  |  |  |  |
| Ortiz et al. (2016) | X | X | X | X | X | X | X | X | X |  | X |  |  | X |  | X |  |  |  |  |  | X |  |  |  |  |  |  |  |  |
| Ortiz et al. (2016) | X | X | X | X | X | X | X | X | X |  | X |  |  | X |  | X |  |  |  |  |  | X |  |  |  |  |  |  |  |  |
| Palomba (2019) | X |  |  | X |  | X |  |  | X |  | X | X |  |  |  | X |  |  |  |  |  |  |  | X |  |  |  |  |  |  |
| Payne et al. (2017) | X | X |  | X | X |  |  | X |  |  | X | X |  | X | X |  | X |  |  |  |  |  |  |  |  |  |  |  |  |  |
| Peveer er al. (2012) | X | X |  | X | X | X | X |  | X | X |  | X | X |  | X | X |  | X | X |  | X | X |  | X |  | X |  |  |  | FLIGHT, TEXT, ADVENTURE |
| Potard et al. (2019) | X | X | X |  | X | X | X | X | X | X |  |  |  |  |  |  |  |  |  |  |  |  |  |  |  |  |  |  |  |  |
| Prena and Sherry (2018) | X | X | X | X | X | X |  |  | X | X |  |  | X |  |  |  |  | X |  | X |  | X |  |  |  |  |  |  |  | EXERGAMES |
| Prevratil et al (2022) | X | X |  |  |  | X |  | X |  |  | X |  |  | X |  |  |  |  |  |  | X |  |  |  |  |  |  |  |  |  |
| Quiroga et al. (2019) | X |  | X |  | X |  |  |  |  |  |  | X | X |  |  |  |  |  |  |  |  |  |  |  |  |  |  |  |  |  |
| Ream et al. (2013) | X | X | X |  |  | X | X | X | X |  |  | X | X |  | X | X |  |  |  |  |  |  |  |  |  |  |  |  |  | X  MOTION CONTROL |
| Rehbein et al. (2016) | X | X |  | X | X | X | X | X |  | X | X |  | X | X |  | X |  | X |  | X | X |  | X | X | X |  | X | X | X | X  BRAIN AND SKILL: QUZ, SILL, FITNESS, HIDDEN OBJECTS, GAME COLLECTION, SIMULATION AND COSNTRUCTION, LIFE SIM, BUSSINESS SIM, PHYSICS, FLIGHT SIM, JUMP ‘N’ RUN, SHOOT ‘EM UP |
| Rodio and Bastien (2013 |  |  |  |  |  |  | X | X |  |  |  |  |  |  | X |  |  |  |  |  |  |  |  |  |  |  |  |  |  |  |
| Salmon et al. (2017) | X | X | X |  | X | X |  | X |  | X |  |  |  |  |  | X |  |  |  |  |  | X |  | X |  |  |  |  |  | FITNESS & LIFESTYLE GAMBLING/CASINO |
| San Nicolas Romera et al. (2018) |  | X |  | X | X |  |  |  | X |  |  | X | X | X | X |  |  |  |  | X |  | X |  |  |  |  | X | X |  | MIXED-NATURE GENRE |
| Scharkow et al. (2015) | X | X | X | X | X |  |  |  |  |  | X | X |  | X |  | X |  |  |  |  |  |  |  |  |  |  |  |  |  |  |
| Seok and DeCosta (2012) |  | X |  |  |  |  |  | X |  |  |  |  |  |  | X |  |  |  |  |  |  |  |  |  |  |  |  |  |  | MULTI USER DUNGEON |
| Schliakhovchuk et al. (2021) | X | X | X | X | X | X |  |  |  |  | X |  | X | X |  |  | X |  |  |  |  |  |  |  |  |  |  |  |  | X  CONSTRUCTION |
| Sjöblom et al. (2017) | X | X |  |  |  |  | X | X | X |  | X |  |  |  | X | X | X | X |  |  |  |  |  |  |  |  |  | X |  |  |
| Stopfer et al.. (2015) |  | X | X | X |  |  |  |  |  |  | X |  |  |  |  |  |  |  |  |  |  |  |  |  |  |  |  |  |  | X |
| Strojny et al.. (2023) | X | X | X | X | X | X | X |  | X | X |  | X | X |  |  |  | X | X |  |  |  |  |  |  |  |  | X |  |  | X  BATTLE ROYALE, |
| Subramanian et al. (2016) |  |  |  |  |  |  | X | X |  |  |  |  |  |  | X |  |  |  |  |  |  |  |  |  |  |  |  |  |  | X |
| Thorne et al. (2014) | X | X |  |  |  | X |  |  |  | X |  |  |  |  |  |  |  |  |  |  |  |  |  |  |  |  |  |  |  |  |
| Upton et al. (2022) | X | X | X | X |  |  | X |  |  | X | X |  |  |  |  |  |  | X | X |  |  |  |  | X |  |  |  |  |  | ADVENTURE, RPG, LOGIC, CASINO, TRIVIA |
| Vahlo and Karhulahti (2020) |  | X | X | X | X | X |  |  |  | X | X | X |  | X |  |  |  |  |  |  |  |  |  |  |  |  |  |  |  |  |
| Vargas-Iglesias (2020) |  |  | X |  |  |  |  |  | X | X |  |  | X |  | X |  | X | X |  |  | X |  |  |  | X | X | X | X | X | FLIGHT SIMULATOR  PINBALL,  SHOOT ’EM’ UP,  FIRST PERSON PUZZLE, GRAPHIC ADVENTURE, INTERACTIVE FICTION., INTERACTIVE MOVIE, DATING SIM, GAMBLING, TURN-BASED TACTICS, LIFE SIMULATOR, REAL-TIME RPG, REAL TIME MMORPG, TURN-BASED RPG, TURN BASED MMORPG, ACTION-PUZZLE, MAZE GAME, REAL-TIME TACTICS, SURVIVAL SANDBOX, TACTICAL SHOOTER, 4X/.BUILT AND BATTLE, STEALTH, TOWER DEFENSE,  RACING SIMULATOR, RT-MMORPG, FREE ROAMING |
| Ventura, Shute, Kim (2012) |  | X | X | X | X |  |  |  | X | X |  | X | X |  |  |  |  |  | X |  |  |  |  |  |  |  |  |  |  |  |
| Vermeulen et al. (2017) | X | X | X |  |  | X | X |  | X | X |  | X | X |  |  | X | X |  | X |  |  |  |  |  |  |  |  |  |  |  |
| Von der Heiden et al. (2019) | X | X | X | X |  | X | X | X |  |  | X |  |  | X | X | X |  | X |  |  | X |  | X |  |  |  |  |  |  | X  TPS, JUMP ‘N’ RUN, SKILL, BRAIN, JOGGING |
| Wohn and Lee (2013) |  | X |  | X |  |  |  |  |  |  |  | X |  |  |  |  |  | X | X |  |  |  |  |  | X |  |  |  |  | WORD GAMES, BRAIN GAMES |
| Yang et al. (2022) | X | X | X | X | X |  |  |  |  |  | X | X |  |  |  |  |  |  |  |  |  |  |  |  | X |  |  |  |  | X  IDLE |
| Yang et al. (2023) | X |  |  |  |  |  | X | X | X |  |  |  |  |  |  |  | X |  |  |  |  |  |  |  |  |  |  |  |  |  |
| Yilmaz, Yel, and Griffiths (2022) | X | X | X | X | X | X |  |  | X | X | X |  |  |  |  |  |  | X |  |  |  | X |  |  |  |  |  |  |  | ENTERTAINMENT, SERIOUS, ROPE |
| Yilmaz, Yel, and Griffiths (2022) | X | X | X | X | X | X |  |  | X | X | X |  |  |  |  |  |  | X | X |  |  | X |  |  |  |  |  |  |  | ENTERTAINMENT, SERIOUS, ELECTRONIC SPORTS, ROPE, LEGO-PUZZLE STONE |
|  | 75 | 75 | 57 | 56 | 55 | 50 | 44 | 43 | 43 | 39 | 39 | 33 | 33 | 30 | 30 | 27 | 27 | 23 | 19 | 14 | 13 | 13 | 9 | 9 | 8 | 8 | 8 | 7 | 5 |  |

*Note*. Individual columns present the genres that occurred in at least 5 analyzed papers. If a given genre was extracted, it was marked with an "X". The last column ('Other') lists genres that have been used less than 5 times.
