# Supplementary material for: The tangled ways to classify games: A systematic review of how games are classified in psychological research
Source: PLoS One. 2024 Jun 24;19(6):e0299819. doi: 10.1371/journal.pone.0299819 (PMC11195997; doi:10.1371/journal.pone.0299819)
Supplement: Attachment — Submitted filename: Response to Reviewers.docx [file pone.0299819.s002.docx]

Response to Reviewer 1

16 December 2023

Dear Dr Mohit Kumar Patralekh,

I am writing this comment in response to the review and suggestions that I received on Novermber 2^nd^, 2023. All of the comments were used to make the manuscript more communicative and meaningful. We would like to thank you for your diligent and constructive review.

Please find the revised version of the manuscript *The tangled ways to classify games: A systematic review of how games are classified in psychological research*. I include the manuscript in marked version.

In the following section you can find detailed comments for your suggestions.

*1. Was the protocol for this systematic review preregistered( on any site like PROSPERO) or pre-published? Please provide details.*

According to PLOS One “Criteria for publication” and PRISMA guidelines, there is no information about obligation of registration. To the best of our knowledge, the preregistrations are recommended more for the research studies than to the reviews. Consequently, our systematic review was not preregistered. The information was added to the manuscript.

*2. Detailed search strategy for at lease one database may be provided.*

The section of search strategy has been transformed to present detailed information about the selected databases, the filters applied regarding the subject, keywords and the selection process. Also described are the exclusion criteria, which previously only appeared on the PRISMA diagram.

*3. The methods section says "The first reviewer screened ScienceDirect and Taylor and Francis records, whereas the second reviewer focused on Scopus and PubMed records." Usually all databases are searched by two reviewers and articles are also selected by both of them, and any discrepancy is resolved by third reviewer. The manuscript is actually silent on whether screening and selection was done by two reviewers.*

Thank you for a very significant comment. In order to eliminate potential bias of independent decision of selection, we conducted a cross-screening which meant re-screening the database results by another reviewer. As a consequence, the databases were screened twice, systematically and independently by two reviewers. In case of discrepancies, they were resolved through clarification and consensus. This information has been added to the method description in the search strategy section.

We would like to inform you that we have updated the literature review for an additional year, extending the coverage from 2012-2022 to 2012-2023. This enhancement ensures that the review is as current as possible and will prove valuable to its audience.

If further improvement is needed, I am at your disposal.

With the best regards,

Patrycja Kiszka on behalf of the authors

Response to Reviewer 2

16 December 2023

Dear Prof Eyüp Yılmaz,

I am writing this comment in response to the review and suggestions that I received on Novermber 2^nd^, 2023. All of the comments were used to make the manuscript more communicative and meaningful. We would like to thank you for your diligent and constructive review, and we also appreciate pointing out specific pieces of literature that we should have included.

Please find the revised version of the manuscript *The tangled ways to classify games: A systematic review of how games are classified in psychological research*. I include the manuscript in marked version.

In the following section you can find detailed comments for your suggestions.

*1. I wonder why the researchers did not include the terms "digital game" and the adjacent "videogame" in their search strategies. Because although the term "video game" is predominantly preferred, we also encounter the use of "digital game" and "videogame" in the literature. This not only leads to the exclusion of studies that meet the criteria but also reduces the content validity of the research. For example, Is there any possibility that the study below was not encountered for this reason and could not be included in the manuscript? Because it seems, the videogames were categorized by their genres as well as traditional games in the study. "Yılmaz, E., Yel, S., & Griffiths, M. D. (2022). Comparison of children's social problem-solving skills who play videogames and traditional games: A cross-cultural study. Computers & Education, 187, 104548.*

In fact, the study (Yilmaz el al., 2022) was not included in the review because of the keyword “video games” with spacebar. According to your comment, for this reason we may have missed relevant studies. Therefore, we decided to conduct a complementary search with the following keywords: “video game genre” OR “video game classification” OR “video game categories” OR “videogame genre” OR “videogame classification” OR “videogame categories” OR “digital game genre” OR “digital game classification” OR “digital game categories”. Additionally, we agreed to expand the temporal frame of the search to include publications from 2023. By doing so, we maximize the chances that all relevant and up-to-date articles are included.

*2. It appears that the databases were searched separately by two different researchers and that a single researcher decided on the studies to be included. There is a problem here in terms of coder reliability. Each database should have been screened by at least two researchers in case of an oversight or indecision, and the procedure followed in case of disagreements should have been explained.*

This note is similar with reviewer 1 and we found those very relevant. In order to eliminate potential bias of independent decision of selection, we conducted a cross-screening which meant re-screening the database results by another reviewer. As a consequence, the databases were screened twice, systematically and independently by two reviewers. In case of discrepancies, they were resolved through clarification and consensus. This information has been added to the method description in the search strategy section.

*3. The researchers need to add not only years but also months to the period (i.e. January 2012 to December 2022 (inclusive)).*

We agree. We have completed the detailed information regarding the time range considered for the search. We would like to inform you that we have updated the literature review for an additional year, extending the coverage from 2012-2022 to 2012-2023. This enhancement ensures that the review is as current as possible and will prove valuable to its audience. This is stated in the first point of the inclusion criterion: “The articles were published between January 2012 and December 2023 (inclusively).”

*4. The results are given within a certain logical framework. However, it would be more systematic if these results were given within the research hypotheses/questions.*

We have taken this suggestion into account through amendments. According to the aim of the study which includes capturing the most recent and the most prominent methods of classifying video game genres in empirical studies, we decided to move the section “Quality of creating game taxonomies” to the top of the results. Then, we provided the parts: ”General characteristics of taxonomies”, “One genre, multiple definitions”, “The multi-genre misunderstanding”, “Steam tags – different approach to game classification”.

*5. Table 1 presents information on the proportion of female participants as well as countries. However, while a detailed discussion addressing the female proportion was made in the discussion section, the same was not done for countries. In this case, I can not sure what the purpose of collecting and publishing data on countries was.*

Indeed, with the large amount of information, we overlooked the discussion of the geographic coverage of the included studies. This section has been supplemented in the manuscript.

*6. I believe the limitations of the manuscript should also have been expressed by the researchers.*

Thank you for the suggestion, we agree. We have added the "Limitations" section to our manuscript.

If further improvement is needed, I am at your disposal.

With the best regards,

Patrycja Kiszka on behalf of the authors
