# Supplementary material for: The tangled ways to classify games: A systematic review of how games are classified in psychological research
Source: PLoS One. 2024 Jun 24;19(6):e0299819. doi: 10.1371/journal.pone.0299819 (PMC11195997; doi:10.1371/journal.pone.0299819)
Supplement: Attachment — Submitted filename: Response to Reviewers.docx [file pone.0299819.s003.docx]

Response to Reviewer 1

11 February 2024

Dear Dr Mohit Kumar Patralekh,

I am writing this comment in response to the review and suggestions that I received on February 5^th^, 2024. We appreciate your feedback and suggestion regarding the mention of the search period in the manuscript. Upon reconsideration, we agree that it would be more appropriate to specify the dates when the last search was performed rather than stating a full year. We will make the necessary adjustments to accurately reflect the timing of the search process in the revised manuscript.

Please find the revised version of the manuscript *The tangled ways to classify games: A systematic review of how games are classified in psychological research*. I include the manuscript in marked version.

If further improvement is needed, I am at your disposal.

With the best regards,

Patrycja Kiszka on behalf of the authors

Response to Reviewer 2

11 February 2024

Dear Prof Eyüp Yılmaz,

I am writing this comment in response to the review and suggestions that I received on February 5^th^, 2024. Thank you for your thorough review and acknowledgment of our efforts in addressing the corrections. Indeed, we concur with your observation. The oversight regarding the inclusion of the "constant" variable has been rectified, and both the manuscript and the supplementary materials have been amended accordingly. We appreciate your keen attention to detail, which has undoubtedly enhanced the quality and integrity of our work.

Please find the revised version of the manuscript *The tangled ways to classify games: A systematic review of how games are classified in psychological research*. I include the manuscript in marked version.

If further improvement is needed, I am at your disposal.

With the best regards,

Patrycja Kiszka on behalf of the authors
